# In Search of an Ideal Test for Diagnosis and Prognosis of Kala-azar

**DOI:** 10.3329/jhpn.v28i3.5557

**Published:** 2010-06

**Authors:** Dharmendra P. Singh, Rahul K. Goyal, Rahul K. Singh, S. Sundar, Tribhuban M. Mohapatra

**Affiliations:** ^1^ Department of Microbiology; ^2^ Department of Medicine, Institute of Medical Sciences, Banaras Hindu University, Varanasi 221005, India

**Keywords:** Diagnosis, Laboratory, Direct agglutination test, KAtex, rK39 strip test, Visceral leishmaniasis, India

## Abstract

The latex agglutination test (KAtex), direct agglutination test (DAT), and the rK39 immuno-chromatographic strip test (dipstick test) were evaluated for their role in the diagnosis and prognosis of visceral leishmaniasis (kala-azar) in India. Sera and urine samples from 455 subjects—150 confirmed visceral leishmaniasis cases, 160 endemic controls, 100 non-endemic controls, and 45 other febrile diseases—were included in the study. The sensitivity of the KAtex, DAT, and rK39 strip test was 87% [95% confidence interval (CI) 80–96], 93.3% (95% CI 88–100), and 98% (95% CI 93–100) respectively. The specificity of these tests was 98% (95% CI 93–100), 93% (95% CI 87–100), and 89% (95% CI 82–97) for the KAtex, DAT, and rK39 strip test respectively. Fifty cases were followed up and subjected to the KAtex, DAT, and rK39 strip test after 30 days of successful treatment. The DAT and rK39 strip test showed positive results in all the 50 cases whereas the KAtex showed no positive reaction in any case. Based on the results, it is concluded that the sensitivity and specificity of the DAT and rK39 strip test are comparable but the greater convenience of use of the strip test makes it a better tool for the diagnosis of visceral leishmaniasis in the peripheral areas of endemic regions whereas the sensitivity of the KAtex needs to be improved to promote its use as a first-line diagnostic test in the field-setting. It may be used for the prognosis of the disease as antigen becomes undetectable in urine after 30 days of the completion of the treatment. Alternatively, it can be used as an adjunct with rK39 for sero-epidemiological surveys.

## INTRODUCTION

Leishmaniasis is endemic in 88 countries spread over five continents, with an estimated yearly incidence of 1–1.5 million cases of cutaneous leishmaniasis and 500,000 cases of visceral leishmaniasis (VL). The number of people at risk is estimated to be 350 million, with an overall prevalence among 12 million people (1). VL is a potentially-fatal disease, caused by *Leishmania donovani* complex. Of all the VL cases, more than 90% are from India, Nepal, Bangladesh, southern Sudan, and northeast Brazil.

For decades, the detection of the parasite has been considered to be the definitive diagnosis of this disease. It requires invasive procedure to find the parasite in the tissue, i.e. spleen, bone-marrow, and lymph-node. Although it is the gold standard for the diagnosis of VL, the procedure is cumbersome, time-consuming, technically demanding, risky, and very difficult to apply in field conditions or remote places/primary health centres. To obviate these procedures, various serological tests have been developed, evaluated, and tried. These tests have the advantage of being safe, are less invasive, and can be carried out in large numbers of samples. Conventional methods of antibody detection are immunodiffusion, complement fixation test, indirect haemagglutination test, and countercurrent immunoelectrophoresis (2–4). However, aside from practical difficulties at peripheral laboratories, the sensitivities and specificities of most of the above tests have been the limiting factors.

Recently, quest for a simple diagnostic test for VL has led to the development of several novel tests, such as direct agglutination test (DAT), rK39 dipstick assay, and latex agglutination test (KAtex) of urine. In particular, the development of different formats of DAT and its subsequent improvement has been an impetus to the improvement of diagnostic procedures of VL under field conditions (5–7). DAT is a sensitive test and well-adapted to screening larger populations, especially when using its variant referred to as fast agglutination screening test (8). However, data regarding its specificity in different populations and patient-groups are variable, and many users of the test find it complicated to perform in remotely-located health facilities.

The rK39 dipstick assay—which is based on a cloned recombinant antigen of 39 amino acid repeats of a kinesin-like gene (9, 10)—and the KAtex of urine latex agglutination test (11) have emerged as attractive options owing either to their simplicity (in the case of rK39 dipstick) or their high specificity (in the case of KAtex). A comparative evaluation of DAT, KAtex, and rK39 dipstick test was conducted aiming at obtaining more reliable estimates of their sensitivity, specificity, and reproducibility in the field for the diagnosis and prognosis of VL in India.

## MATERIALS AND METHODS

Patients were recruited at the Kala-azar Medical Research and Training Centre (KAMRC) in Muzaffarpur and Rajendra Memorial Research Institute (RMRI), Patna, Bihar, India. All clinically-suspected VL cases were subjected to parasitological examination. In total, 455 subjects—comprising 150 confirmed VL cases, 160 endemic controls, and 100 non-endemic controls, and 45 other febrile diseases—were included in the study. Sera and urine samples were collected. The cases were bled on the first encounter. The serum was separated and stored at -20 ºC till further use. However, the KAtex was done in fresh urine samples. The tests were carried out in duplicate in each patient's sample with known positive and negative controls by a technical expert who had no knowledge about the patient's data.

### Reference standard

The cases presented with clinical symptoms and signs suggestive of kala-azar, i.e. fever of two weeks or more, splenomegaly (8.4±2.4 cm), and anaemia. Routine investigations were carried out to exclude other aetiologies of fever with hepato-splenomegaly. The final diagnosis of VL was established by Karnofsky score, white blood cell (WBC) count, haemoglobin assay, and demonstration of parasites in splenic aspirates.

Non-endemic controls included healthy persons from Europe and North America where kala-azar is not endemic. Endemic controls included healthy persons free of signs and symptoms of any disease, residing in same endemic area and are closely related to VL patients.

### Latex agglutination test

The KAtex kit made in the USA was provided by Kalon Biological Limited (Sitting Bourne ME 98 AQ, UK)—lot no. K1-22 and Catalogue no. 5050-001. The test was conducted as per instructions of the manufacturers. Briefly, all reagents were brought to ambient temperature. The test latex was shaken well before use. Fifty mL of urine samples were heated by boiling for five minutes. Heat-treated urine samples were first allowed to cool to ambient temperature and then transferred to a glass-slide. Subsequently, the processed sample was mixed with a drop of test latex and stirred. The glass-slides were continuously rotated for two minutes before final reading of reactions. Positive reactions were determined by the degree of agglutination and recorded as 1+, 2+, and 3+. Appropriate positive and negative controls, supplied by the manufacturer, were included as test control.

### Direct agglutination test

*L. donovani* (MHOM/IN/80/Dd8) obtained from the World Health Organization reference centre was used as antigen and prepared as per the method of Goyal *et al.* (12). Serum samples were serially two-fold diluted with saline and 0.2% gelatin solution. Fifty mL of parasites suspension was added to an equal volume of diluted serum in each well. After the completion of the test, microtitre plates were shaken on a flat and smooth surface and were incubated at 22 °C overnight. The results were recorded in titres. The positive result was expressed as the highest dilution of serum that showed definite agglutination of parasite. Uniform mat formation suggested a positive result. A sample was considered positive if it had a titre of ≥1:3200 (cut-off value).

### rK39 dipstick

The kit was provided by *In Bios* International, Inc. Seattle, Washington, DC, USA. The test was conducted according to instructions of the manufacturer. The strip for VL assay is a qualitative membrane-based immunoassay for the detection of antibodies to VL in human serum. In brief, 20 μL of serum was placed on pad. Following this, 2–3 drops of wash buffer (provided) were added. The mixtures were carried up by capillary action of the membrane VL. The result was read within 10 minutes. The appearance of two pink lines—one control and one test—indicated a positive result while the appearance of one pink line (control) indicates that the test methodology is correct but sample is negative for antibodies.

### Followed up cases

All the patients were provided a standard treatment with amphotericin B (Sarabhai Chemicals, Vadodara, India) in 1 mg/kg infusions on alternate days up to 15 infusions. After the completion of treatment, the patients were free of symptoms. The haematological parameters were restored. Fifty easily accessible (those who reported) among treated patients (n=150) were further followed up. Serum and urine samples were collected to know the prognostic value of the rK39 dipstick test, DAT, and KAtex.

### Statistical analysis

The serological data, thus, obtained were analyzed after entry in the Epi Info software (version 6) and the SPSS package (version 16). The Fleiss quadratic method was used in calculating the confidence interval of proportions. The sensitivity and specificity were mathematically calculated as follows: sensitivity=(true positive/true positive+false negative)x100; Specificity=(true negative/true negative+false positive)x100.

### Ethics

The Ethical Committee of the Institute of Medical Sciences, Banaras Hindu University, approved the study. Written consent was obtained from all the subjects.

## RESULTS

Of the 150 parasitologically-proven index cases, 131 (87%), 140 (93.3%), and 148 (98%) were positive for the KAtex, DAT, and rK39 strip test respectively. Of the healthy controls (n=160), 2 (1.2%), 5 (3.1%), and 17 (10.6%) showed positive reaction for the KAtex, DAT, and rK39 strip test respectively while none from the non-endemic area showed positive reaction. No cross reactivity was found between the VL cases and the other febrile cases. The table shows the results.

**Table.** Comparison of KAtex, DAT, and rK39 strip test in patients with visceral leishmaniasis and controls

Clinical status	No.	KAtex-positive		DAT-positive		rK39 strip test-positive	
		No.	%	No.	%	No.	%
Confirmed VL	150	131	87	140	93.3	148	98
Non-endemic controls	100	0	0	0	0	0	0
Endemic controls	160	2	1.2	5	3.1	17	10.6
Other diseases	45	0	0	0	0	0	0

DAT=Direct agglutination test;

KAtex=Latex agglutination test;

VL=Visceral leishmaniasis

**Fig. 1.**
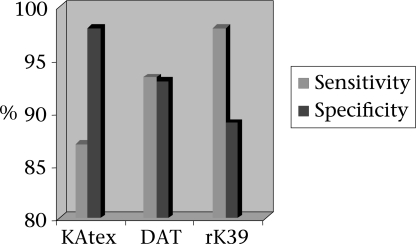
Diagnostic parameter of KAtex, DAT, and rK39 strip test

**Fig. 2.**
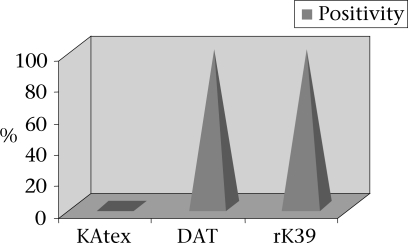
Comparative evaluation of KAtex, DAT, and rK39 strip test in post-treated cases

The sensitivity of the KAtex, DAT, and rK39 strip test was 87% (95% CI 80–96), 93.3% (95% CI 88–100), and 98% (95% CI 93–100) respectively. The specificity of these tests was 98% (95% CI 93–100), 93% (95% CI 87–100), and 89% (95% CI 82–97) for the KAtex, DAT, and rK39 strip test respectively (Fig. 1). Fifty clinically-cured cases were followed up and subjected to the KAtex, DAT and rK39 strip test after 30 days of successful treatment. The DAT and rK39 strip test showed positive results in all the 50 (100%) cases whereas the KAtex showed no positive reaction in any case (Fig. 2).

DAT=Direct agglutination test;
KAtex=Latex agglutination test

DAT=Direct agglutination test;
KAtex=Latex agglutination test

## DISCUSSION

The present study was conducted on 455 subjects for the field evaluation of the KAtex, DAT and rK39 strip test as a diagnostic and prognostic tool for kala-azar. In this study, the DAT and rK39 strip test were evaluated on serum because the tests were carried out in one-go. In clinical practice, however, these tests may be performed on whole fingerprick blood. In our study, the KAtex showed 87% (95% CI 80–96) sensitivity. These results corroborated well with the results of other researchers (13, 14) who showed a sensitivity in the range of 87–95% where fresh urine samples were tested. However, the results do not corroborate with those of other studies (15–17) where poor sensitivity has been reported, which may be due to low level of parasitaemia at a particular stage of disease or may be attributed to the effect of transport and subsequent storage of urine. Poor-to-moderate reproducibility of the KAtex mentioned by other researchers (15, 16) is an additional disadvantage of the test. On the other hand, our results on the specificity of the KAtex are consistent with those of previous studies which ranged from 98% to 100% (14–16). The positive KAtex by 1.2% (2 of 160) in the endemic controls points out to subclinical infection, having a low level of parasitaemia. Subclinical VL with a few or no symptoms with positive anti-leishmanial serology is documented (9). In this study, cut-off titre of ≥1:3,200 was employed for the DAT. Our results showed the sensitivity and specificity of 93.3% and 93% respectively. These results corroborated well with the results of other researchers (18–23). The rK39 strip test showed 98% sensitivity and 89% specificity. It showed negative reaction with the non-endemic controls whereas 10.6% of the subjects from an endemic area were positive by the rK39 strip test; 10.6% positivity in the endemic controls but negative reaction with the non-endemic controls indicates false-positive reaction. False-positivity with serological tests is significantly higher than with the antigen test (KAtex). On the contrary, all the febrile cases showed non-reactive results by this method. This result corroborated well with the finding of other researchers (24–26). The specificity of the test is lower because of the higher prevalence of subclinical or past infections. On the contrary, the study in Sudan showed lower sensitivity and specificity of this test (27). The reason for this difference in antibody response may be because of the ethnic differences or the difference in antibody response.

In the present study, 50 successfully-treated individuals were followed up to evaluate the prognostic value of the KAtex, DAT, and rK39 strip test after 30 days of the completion of treatment. All showed positive reaction for the rK39 strip test and DAT, which indicates persistence of antibodies whereas the KAtex showed no positive reaction, indicating absence of antigens in urine. These observations were supported in various studies performed in successfully-treated cases (26, 28–30).

If we compare the overall yield of these diagnostic tests, the DAT and rK39 strip test are comparable. The advantage of DAT includes the availability of titres; however, the procedure for the DAT is cumbersome and requires incubation, and the storage of antigen also requires a cold-chain. This may not be possible in remote rural India. By contrast, the rK39 strip test procedure is easy, simple, and user-friendly and can be easily performed and interpreted in most peripheral areas even by an auxiliary health worker. Thus, the rK39 strip test has the potential to be used as a suitable candidate for the diagnosis of VL whereas the prognostic value of the KAtex seems to be promising.

The study was carried out only in a limited number of post-treated kala-azar cases. For the correct assessment of these tests, more number of cases with well-matched controls may be carried out to reach a definite conclusion. However, with these observations, it may be concluded that the rK39 strip test might be used as a diagnostic tool for kala-azar. Since this is a highly-sensitive test, an ideal format for use under field conditions, quick and no special equipment is needed. For monitoring successful treatment, the KAtex may be used because antigen becomes undetectable in urine after successful treatment. For sero-epidemiological studies, both rK39 and KAtex may be carried out. Their simplicity and detection of both antigen and antibodies may give better information on the disease prevalent in a particular area.

## ACKNOWLEDGEMENTS

The authors express their gratitude to Dr. S.K. Kar, former Director of KAMRC, Patna, for providing the patients and the facilities to carry out the DAT and KAtex.

## References

[1] Desjeux P (2001). The increase in risk factors for leishmaniasis worldwide. Trans R Soc Trop Med Hyg.

[2] Bray RS, Cohen S, Sadun EH (1976). Immunodiagnosis of leishmaniasis. Immunology of parasitic infections.

[3] Hockmeyer WT, Wellde BT, Sabwa CL, Smith DH, Rees PH, Kager PA (1984). A complement fixation test for visceral leishmaniasis using homologous parasite antigen I. Ann Trop Med Parasitol.

[4] Aikat BK, Sehgal S, Mahajan RC, Pathania AG, Bhattacharya PK, Sahaya S (1979). The role of counter immunoelectrophoresis as a diagnostic tool in kala-azar. Indian J Med Res.

[5] Harith AE, Kolk AH, Kager PA, Leeuwenburg J, Muigai R, Kiugu S (1986). A simple economical direct agglutination test for serodiagnosis and sero-epidemiological studies of visceral leishmaniasis. Tran R Soc Trop Med Hyg.

[6] el Harith A, Kolk AH, Leeuwenburg J, Muigai R, Huigen E, Jelsma T (1988). Improvement of direct agglutination test for field studies of visceral leishmaniasis. J Clin Microbiol.

[7] Meredith SE, Kroon NC, Sondorp E, Seaman J, Goris MG, van Ingen CW (1995). Leish-KIT, a stable direct agglutination test based on freeze-dried antigen for serodiagnosis of visceral leishmaniasis. J Clin Microbiol.

[8] Hailu A, Kroon CC, Schoone GJ, Berhe N, Schallig HD, Kager PA (2002). Sero-epidemiological assessment and diagnosis of visceral leishmaniasis in an endemic locality using the Fast Agglutination Screening Test (FAST). Acta Tropica.

[9] Badaró R, Benson D, Eulálio MC, Freire M, Cunha S, Netto EM (1996). rK39: a cloned antigen of *Leishmania chagasi* that predicts active visceral leishmaniasis. J Infect Dis.

[10] Burns JM, Shreffler WG, Benson DR, Ghalib HW, Badaro R, Reed SG (1993). Molecular characterization of a kinesin-related antigen of *Leishmania chagasi* that detects specific antibody in African and American visceral leishmaniasis. Proc Natl Acad Sci USA.

[11] Attar ZJ, Chance ML, el-Safi S, Carney J, Azazy A, El-Hadi M (2001). Latex agglutination test for the detection of urinary antigens in visceral leishmaniasis. Acta Trop.

[12] Goyal RK, Mohapatra TM (2004). Superiority of DAT over ELISA as a diagnostic and seroepidemiological tool for the diagnosis of Indian kala-azar. Ind J Med Microbiol.

[13] Sundar S, Agrawal S, Pai K, Chance M, Hommel M (2005). Detection of leishmanial antigen in the urine of patients with visceral leishmaniasis by a latex agglutination test. Am J Trop Med Hyg.

[14] El-Safi SH, Abdel-Haleem A, Hammad A, El-Basha I, Omer A, Kareem HG (2003). Field evaluation of latex agglutination test for detecting urinary antigens in visceral leishmaniasis in Sudan. East Mediterr Health J.

[15] Chappuis F, Rijal S, Jha UK, Desjeux P, Karki BM, Koirala S (2006). Field validity, reproducibility and feasibility of diagnostic tests for visceral leishmaniasis in rural Nepal. Trop Med Int Health.

[16] Rijal S, Boelaert M, Regmi S, Karki BM, Jacquet D, Singh R (2004). Evaluation of a urinary antigen-based latex agglutination test in the diagnosis of kala-azar in eastern Nepal. Trop Med Int Health.

[17] Sundar S, Singh RK, Bimal SK, Gidwani K, Mishra A, Maurya R (2007). Comparative evaluation of parasitology and serological tests in the diagnosis of visceral leishmaniasis in India: a phase III diagnostic accuracy study. Trop Med Int Health.

[18] Aoun K, Bouratbine A, Chahed MK, Ben Ismail R (2000). Role of direct agglutination test (DAT) in the diagnosis of viscearal leishmaniasis in Tunisia. Tunis Med.

[19] Boelaert M, El Safi S, Jacquet D, de Muynck A, van der Stuyft P, Le Ray D (1999). Operational validation of the direct agglutination test for diagnosis of visceral leishmaniasis. Am J Trop Med Hyg.

[20] Garcez LM, Shaw JJ, Silveira FT (1996). [Direct agglutination tests in the serodiagnosis of visceral leishmaniasis in the state of Pará]. Rev Soc Bras Med Trop [Portuguese]..

[21] Singla N, Singh GS, Sundar S, Vinayak VK (1993). Evaluation of the direct agglutination test as an immunodiagnostic tool for kala-azar in India. Trans R Soc Trop Med Hyg.

[22] Boelaert M, El-Safi S, Hailu A, Mukhtar M, Rijal S, Sundar S (2008). Diagnostic tests for kala-azar: a multi-centre study of the freeze-dried DAT, rK39 strip test and KAtex in East Africa and the Indian subcontinent. Trans R Soc Trop Med Hyg.

[23] Chappuis F, Rijal S, Soto A, Menten J, Boelaert M (2006). A meta-analysis of the diagnostic performance of the direct agglutination test and rK39 dipstick for visceral leishmaniasis. BMJ.

[24] Sundar S, Reed SG, Singh VP, Kumar PC, Murray HW (1998). Rapid accurate field diagnosis of Indian visceral leishmaniasis. Lancet.

[25] Singh S, Kumari V, Singh N (2002). Predicting kala-azar disease manifestations in asymptomatic patients with latent *Leishmania donovani* infection by detection of antibody against recombinant K39 antigen. Clin Diagn Lab Immunol.

[26] Singh DP, Sundar S, Mohapatra TM (2009). The rK39 strip test is non-predictor of clinical status for kala-azar. BMC Res Notes.

[27] Zijlstra EE, Nur Y, Desjeux P, Khalil EA, El-Hassan AM, Groen J (2001). Diagnosing visceral leishmaniasis with the recombinant K39 strip test: experience from the Sudan. Trop Med Int Health.

[28] Zijlstra EE, Daifalla NS, Kager PA, Khalil EA, El-Hassan AM, Reed SG (1998). rK39 enzyme-linked immunosorbent assay for diagnosis of *Leishmania donovani* infection. Clin Diagn Lab Immunol.

[29] Kumar D, Srividya G, Verma S, Singh R, Negi NS, Fragaki K (2008). Presence of anti-Lepp12 antibody: a marker for diagnostic and prognostic evaluation of visceral leishmaniasis. *Trans*. R Soc Trop Med Hyg.

[30] Gavagni AS, Vatan SK, Ghazanchaei A (2008). KAtex antigen detection test as a diagnostic tool for latent visceral leishmaniasis cases. Afr J Biotechnol.

